# Nutritional Indicators for Gujarat, Its Determinants and Recommendations: A Comparative Study of National Family Health Survey-4 and National Family Health Survey-5

**DOI:** 10.7759/cureus.39175

**Published:** 2023-05-18

**Authors:** Jimeet Soni, Faisal S Sheikh, Somen Saha, Mayur B Wanjari, Deepak Saxena

**Affiliations:** 1 Public Health, Indian Institute of Public Health Gandhinagar, Gandhinagar, IND; 2 Research Scientist, Jawaharlal Nehru Medical College, Datta Meghe Institute of Medical Sciences, Wardha, IND

**Keywords:** anemia, gujarat, determinants, malnutrition, nutrition indicators

## Abstract

Malnutrition is a public health problem globally. Gujarat is one of the states facing challenges in dealing with malnutrition and anemia. The NFHS-5 (National Family Health Survey-5) data reveals that the gains made in NFHS-4 (National Family Health Survey-4) were reversed in NFHS-5. Despite numerous schemes and policies in place, Gujarat has yet to reach the full potential of these mandated policies to showcase exponential results in malnutrition and anemia. This study presents an overview of the nutritional status of districts in Gujarat, compared with NFHS-4, by illuminating its potential determinants and inter-district variabilities. An increased prevalence was seen in children under five who are stunted and severely wasted; however, the prevalence of children under five who are wasted improved in Gujarat. The prevalence of anemia increased across all age groups, showing an immediate sign of caution. The study observed decreased prevalence of immediate determinants and increased coverage of nutrition-specific interventions in NFHS-5 compared to NFHS-4 for nutritional indicators in Gujarat. Underlying determinants like households with electricity and improved drinking water have improved drastically in Gujarat. Furthermore, it elaborates on the gaps and improvements observed in inter-district variabilities among determinants in their coverage. This study also consists of actions taken by states that have fared better concerning nutritional indicators instead of improving the nutritional indicators for Gujarat. The study has categorized the districts into top-priority, priority, average, and front-runner districts of Gujarat based on the prevalence of nutritional indicators.

## Introduction and background

Malnutrition among children under five years in India is a major public health concern [1]. There is ample amount of evidence that has highlighted the serious implications that malnutrition among children carries with itself on a child’s development. It has an adverse impact on physical as well as cognitive development, in turn affecting the overall productivity as well as economic development of the nation [2]. The world bank has stated an adverse loss of 1.4% in economic fruitfulness due to childhood stunting, demonstrating a 1% loss in adult height [3]. The prevalence of malnutrition is the highest in the world and is almost double that of Sub-Saharan Africa [4,5]. Besides malnutrition, Anemia has remained an important global health issue [6]. Anemia affects over 50% of adolescent girls, particularly from low socio-economic backgrounds, and the findings show little improvement in 10 years [7]. Anemia has various correlates based on its underlying pathophysiology, nutritional deficiencies, and chronic diseases [8,9]. Anemia in women is a well-known cause of poor fetal growth and low birth weight [2]. One in every two women who enter into pregnancy is anemic. They report to antenatal clinics around eight to 10 weeks of gestation, which can prove untimely for improving or impacting birth weight and length [10]. With an increased understanding of malnutrition's multifactorial causes and complexity, there is a compelling need to adopt and promote a multisectoral approach to achieve better nutrition outcomes.

The National Family Health Survey (NFHS) is a large-scale multi-round survey conducted in a representative sample of households to provide national, state/union territory (UT), and district-level estimates of various indicators all over India. NFHS-5 provides district-level estimates for many important indicators [11]. Nutrition is an important determinant of a healthier population, while malnutrition in all its forms is a vital risk factor with serious implications on morbidity across the course of life. This report aims to provide substantial data about the nutritional indicators, namely the nutritional status of children; the nutritional status of adults (age 15-49 years); anemia among children, adults, men, pregnant, and non-pregnant women, and identify high-priority districts in the aforementioned indicators along with determinants affecting these indicators.

## Review

Methods

We utilized summary data from the recently released NFHS-5, 2019-2020, and compared it with NFHS-4, 2015-2016, Gujarat state and district fact sheets by the International Institute of Population Science [12-17]. For outcome indicators, the progress on a set of global nutrition targets for maternal, infant, and young child nutrition was examined, which includes stunting, wasting, severe wasting, and anemia status among women of reproductive age. We also examined changes in several immediate and underlying determinants of nutrition. However, we observe the change in nutrition-specific interventions across the life cycle for intervention coverage. All the figures and maps were prepared using NFHS-4 and NFHS-5 data.

Trends

The latest NFHS-5 data suggests that stunting among children below five years lacked improvement. More than half of the states/UTs have reported that every third child below five years suffers from stunting. Most of the districts of Bihar and Meghalaya and a few districts of Gujarat were comparatively weaker than other states. In the recent half-decade, acute malnutrition has worsened in most states/UTs, with Nagaland, Manipur, Mizoram, Assam, Telangana, and Bihar experiencing a significant increase. Every fourth child was thin for their age in Bihar, Gujarat, and Maharashtra. Mizoram, Manipur, Meghalaya, Sikkim, and Kerala performed best when tackling acute child malnutrition, whereas Maharashtra, Gujarat, Bihar, Assam, and Telangana performed poorly.

Central India has a higher prevalence of wasting than the rest of the country. Underweight prevalence among children of zero to 59 months remained stagnant in most states/UTs since NFHS-4. Nagaland was found to be having noticeable changes during the last five years. Bihar reported the highest prevalence of underweight children, followed by Gujarat. Most underweight children were seen in various regions of Gujarat, Maharashtra, Karnataka, Bihar, and West Bengal. Five districts of Gujarat reported that more than 50% of children were undernourished. Child obesity increased across all states except Goa, with an increase in the proportion of overweight children under five. Among these, Mizoram, Jammu, and Kashmir reported the highest prevalence, wherein Mizoram and Tripura showed an unambiguous increase in their prevalence. Anemia among children and women remains a continuous cause of concern in 13 states/UTs of India, as more than half of them were anemic. Anemia among pregnant women has increased in half of the states/UTs compared to NFHS-4, despite a substantial increase in the consumption of iron and folic acid (IFA) tablets by pregnant women for 180 days or more.

Trends in nutrition outcomes in Gujarat

The data reveals a substantial drop nationally in the percentage of stunted, wasted, and underweight children under five from NFHS-4 to NFHS-5. In 2015-2016, 38.4% of children below five years were stunted, 21% were wasted, and 35.8% were underweight in India. NFHS-5 data shows that 35.5% of children below five years were stunted, 19.3% were wasted, and 32.1% were underweight, with a reduction in prevalence than NFHS-4. There was a slight increase in the prevalence of severely wasted children from 7.5% in NFHS-4 to 7.7% in NFHS-5 in India. As observed in NFHS-5, the prevalence of overweight children under five was 3.4% in India.

**Figure 1:**
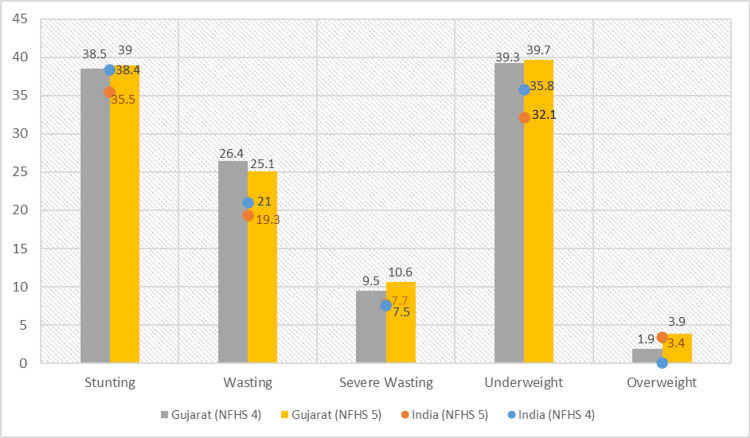
Nutritional status of children under five years The author has adopted the figure from NFHS-4 and NFHS-5 open-source data [11-15]. NFHS, National Family Health Survey

The findings of NFHS-5 of Gujarat state suggested an increase in the prevalence of stunted, severely wasted, underweight, and overweight children under five. Comparatively, the prevalence of stunting has slightly increased from 38.5% to 39%, severe wasting from 9.5% to 10.6%, and underweight from 39.3% to 39.7% in NFHS-5. There is a reduction in wasting from 26.4% to 25.1%, and the prevalence of under-five children who reported being overweight has increased from 1.9% to 3.9% in NFHS-5 (Figure 1).

The comparative data of NFHS-4 and NFHS-5 suggested a considerable reduction in the percentage of women below normal BMI (<18.5 kg/m^2^) from 22.9% to 18.7% and men whose BMI is below normal (<18.5 kg/m^2^) from 20.2% to 16.3% nationally. There was an increment in the prevalence of women who are overweight or obese (BMI ≥25 kg/m^2^) from 20.6% to 24% and men who are overweight or obese (BMI ≥25 kg/m^2^) from 18.9% to 22.9% in India.

**Figure 2:**
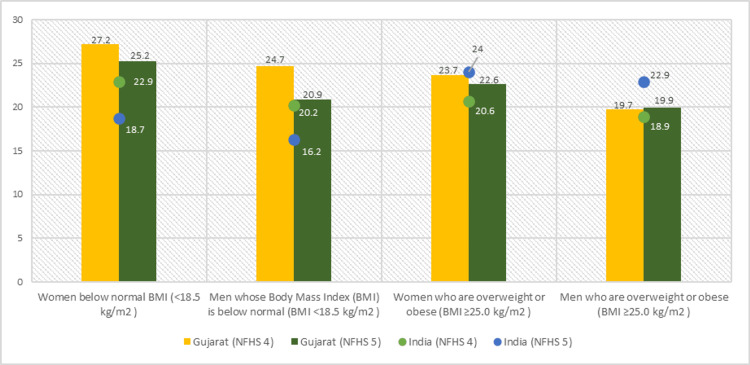
Nutritional status of adults (age 15-49 years) The author has adopted the figure from NFHS-4 and NFHS-5 open-source data [11-15]. NFHS, National Family Health Survey

However, the state of Gujarat fared better in NFHS-5 by having a substantial drop in the prevalence of women below normal BMI (<18.5 kg/m^2^) from 27.2% to 25.2%, men with BMI below normal from 24.7% to 20.9%, overweight/obese women from 23.7% to 22.6% compared to NFHS-4. The prevalence of men who are overweight/obese was seen to be slightly increased from 19.7% to 19.9% (Figure 2).

The prevalence of anemia in NFHS-5 among children aged six to 59 months (<11 gm/dL) increased from 58.6% to 67.1%, increased from 53.2% to 57.2% in non-pregnant women aged 15 to 49 years (<12 gm/dL), increased from 50.4% to 52.2% in pregnant women aged 15 to 49 years (<11 gm/dL), increased from 53.1% to 57% in all anemic women aged 15 to 49 years, and increased from 22.7% to 25% in anemic men aged 15 to 49 years (<13 gm/dL) compared to NFHS-4 in India.

**Figure 3:**
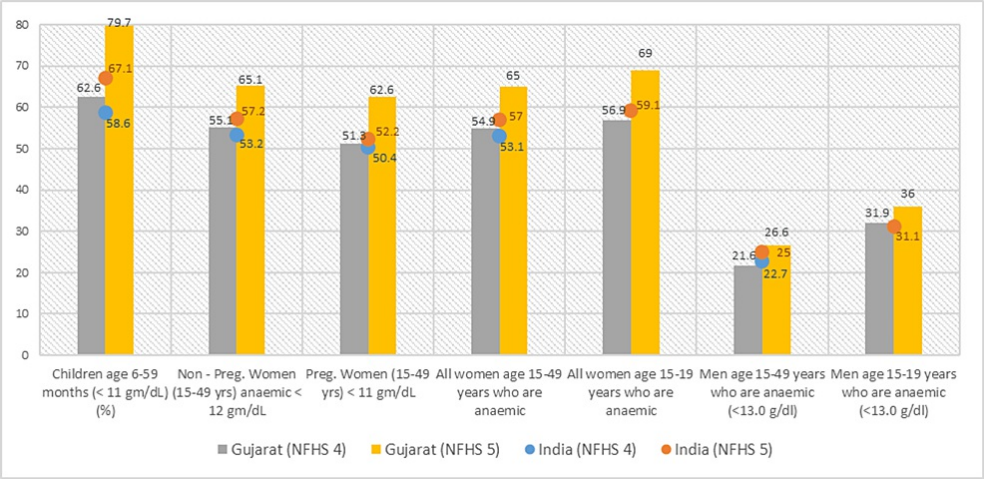
Prevalence of anemia The author has adopted the figure from NFHS-4 and NFHS-5 open-source data [11-15]. NFHS, National Family Health Survey

The data shows an increased trend in the prevalence of anemia in NFHS-5 in Gujarat state compared to NFHS-4. The prevalence of anemia in NFHS-5 among children aged six to 59 months (<11 gm/dL) increased from 62.6% to 79.7%, increased from 55.1% to 65.1% in non-pregnant women aged 15 to 49 years (<12 gm/dL), increased from 51.3% to 62.6% in pregnant women aged 15 to 49 years (<11 gm/dL), increased from 54.9% to 65% in all anemic women aged 15 to 49 years, increased from 56.9% to 69% in all anemic women aged 15 to 19 years, increased from 21.6% to 26.6% in anemic men aged 15 to 49 years (<13 gm/dL), and increased from 31.8% to 36% in anemic men aged 15 to 19 years (<13 gm/dL) compared to NFHS-4 in India (Figure 3).

Gujarat state highlights

**Figure 4:**
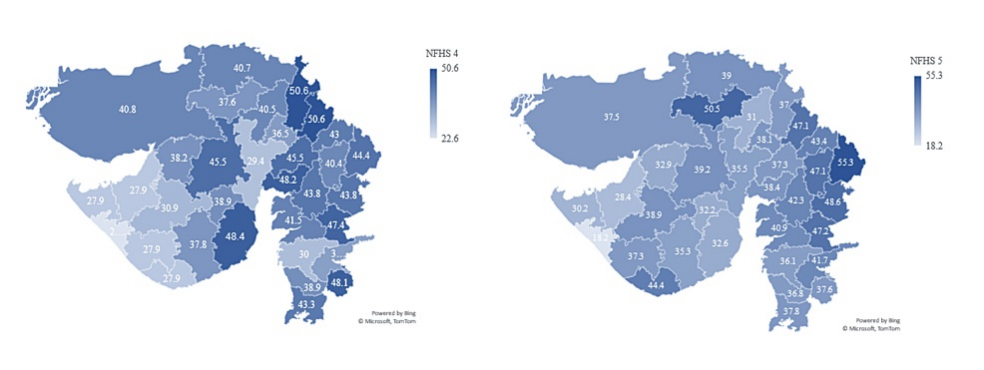
Showing children under five who are stunted (height for age %) The author has adopted the figure from NFHS-4 and NFHS-5 open-source data [11-15]. NFHS, National Family Health Survey

Children under five who are stunted (height for age %) show an increase in state percentage in NFHS-5 compared to NFHS-4, that is, NFHS-4 showed a prevalence of 38.5%, whereas NFHS-5 shows a prevalence of 39%. According to NFHS-5, the most affected districts are Dahod (55.3%), Patan (50.5%), and Chotta Udepur (48.6%), and it is concerning that these districts have deteriorated from NFHS-4. However, on the other hand, districts like Aravalli (47.1% from 50.6%), Bhavnagar (32.6% from 48.4%), and Sabarkantha (37% from 50.6%) have improved drastically in NFHS-5 from NFHS-4. Out of the 33 districts in Gujarat, 13 districts have shown an increase in the prevalence of children under five who are stunted (height for age %). According to NFHS-5, the better-performing districts are Devbhoomi (30.2%), Jamnagar (28.4%), and Porbandar (18.2%). The national mean is 35.5% (NFHS-5), 3.5% better than Gujarat’s prevalence of 39% (NFHS-5) (Figure 4).

**Figure 5:**
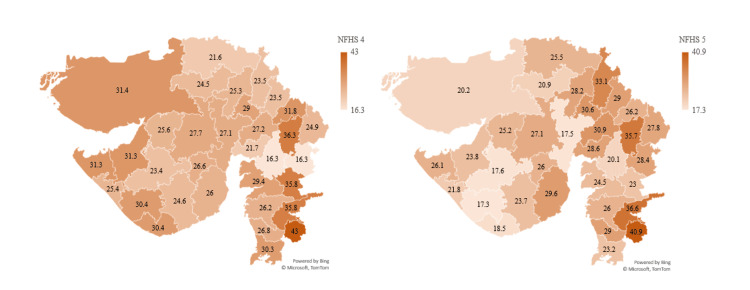
Showing children under 5 who are wasted (weight for height %) The author has adopted the figure from NFHS-4 and NFHS-5 open-source data [11-15]. NFHS, National Family Health Survey

Children under five who are wasted (weight for height %) show a mean decrease in prevalence from 26.4% in NFHS-4 to 25.1% in NFHS-5 in Gujarat. According to NFHS-5, the most affected districts are The Dangs (40.9%), Tapi (36.6%), and Panchmahal (35.7%), while the better-performing districts are Rajkot (17.6%), Ahmedabad (17.5%), and Junagadh (17.3%). A total of nine districts, Anand (from 21.7% to 28.6%), Aravalli (from 23.5% to 29%), Banaskantha (from 21.6% to 25.5%), Bhavnagar (from 26% to 29.6%), Chotta Udepur (from 16.3% to 28.4%), Kheda (from 27.2% to 30.9%), Navsari (from 26.8% to 29%), Sabarkantha (from 23.5% to 33.1%), and Vadodara (from 16.3% to 20.1%), have deteriorated in NFHS-5. While Rajkot, Ahmedabad, and Junagadh have controlled the prevalence in their district below 19%, better than the state average of 25.1% (Figure 5). 

**Figure 6:**
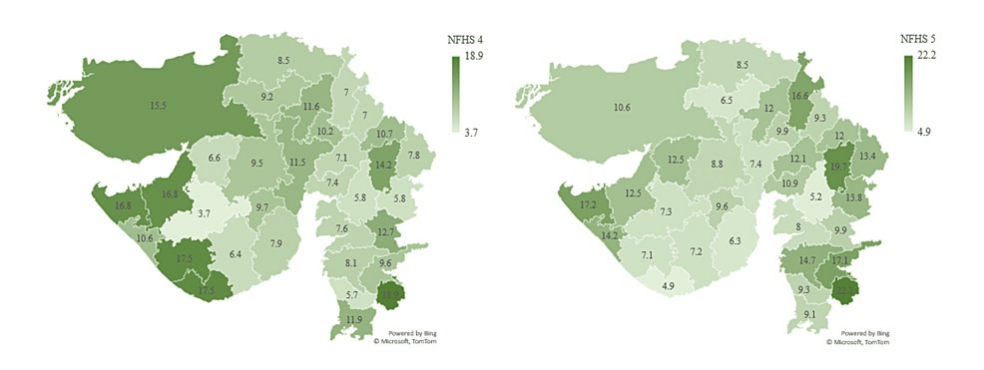
Showing children under five who are severely wasted (weight for height %) The author has adopted the figure from NFHS-4 and NFHS-5 open-source data [11-15]. NFHS, National Family Health Survey

Children under five who are severely wasted (weight for height %) show a mean increase in the prevalence with 10.6% in NFHS-5 compared to 9.5% in NFHS-4 in Gujarat. According to NFHS-5, the most affected districts are The Dangs (22.2%), Panchmahal (19.7%), and Devbhumi Dwarka (17.2%). Better-performing districts are Bhavnagar (6.3%), Vadodara (5.2%), and Gir Somnath (4.9%). A cause of worry is that out of 33 districts, 19 districts have increased prevalence in NFHS-5 compared to NFHS-4. This shows the shocking reality of children under five being severely wasted (Figure 6).

**Figure 7:**
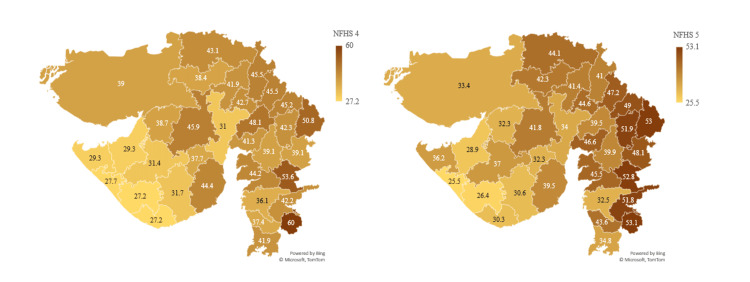
Showing children under five who are underweight (weight for age %) The author has adopted the figure from NFHS-4 and NFHS-5 open-source data [11-15]. NFHS, National Family Health Survey

Children under five who are underweight (weight for age %) show a marginal increase in the prevalence with 39.7% in NFHS-5 compared to 39.3% in NFHS-4 in Gujarat. According to NFHS-5, the most affected districts are The Dangs (53.1%), Dahod (53%), and Narmada (52.8%). The districts that performed better were Jamnagar (28.9%), Junagadh (26.4%), and Porbandar (25.5%). Out of 33 districts, 17 districts have increased prevalence in NFHS-5 compared to NFHS-4. When conjectured with children under five who are severely wasted in NFHS-5 compared to NFHS-4, it can be inferred that more children are slipping from normal to underweight to severe acute malnutrition (SAM) (Figure 7).

**Figure 8:**
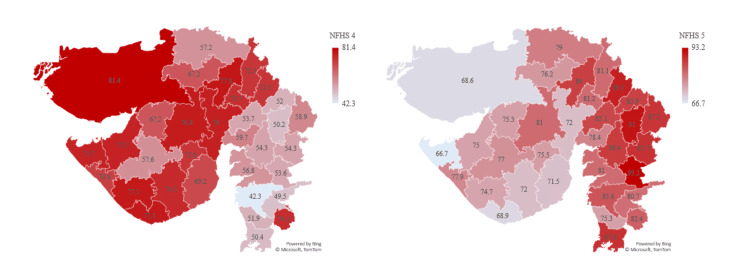
Prevalence of anemia in children aged six to 59 months (<11 gm/dL %) The author has adopted the figure from NFHS-4 and NFHS-5 open-source data [11-15]. NFHS, National Family Health Survey

Prevalence of anemia in children aged six to 59 months (<11 gm/dL %) shows a worrisome increase with 79.7% in NFHS-5 compared to 62.6% in NFHS-4 in the state of Gujarat. According to NFHS-5, the most affected districts are Narmada (93.2%), Panchmahal (91%), and Aravalli (89.5%). The districts that showed lower prevalence were Gir Somnath (68.9%), Kachch (68.6%), and Devbhumi Dwarka (66.7%). A shocking 26 districts of Gujarat show increased prevalence in NFHS-5 compared to NFHS-4 (Figure 8).

**Figure 9:**
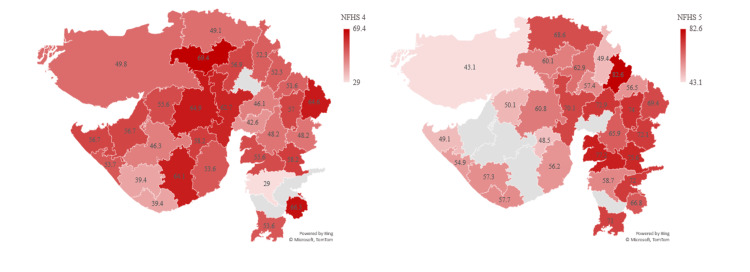
Prevalence of anemia in all women aged 15 to 49 years The author has adopted the figure from NFHS-4 and NFHS-5 open-source data [11-15]. NFHS, National Family Health Survey

All women aged 15 to 49 years who are anemic show a 10% increase in the prevalence of 65% in NFHS-5 compared to 54.9% in NFHS-4 in Gujarat. According to NFHS-5, the most affected districts are Chotta Udepur (78.9%), Tapi (77.4%), and Aravalli (77.3%). The districts that showed lower prevalence were Bhavnagar (49.4%), Devbhumi Dwarka (48.8%), and Porbandar (47.6%). A shocking 23 districts of Gujarat show increased prevalence in NFHS-5 compared to NFHS-4 (Figure 9).

The nutritional status of district-wise under-five children with change in mean from NFHS-4 to NFHS-5 is provided in Table 2 of Appendices, the nutritional status of adults (age 15-49 years) is provided in Table 3 of Appendices, and the prevalence of anemia is provided in Table 4 of Appendices with its mean difference.

Changes in the potential determinants of nutrition

Malnutrition in children is triggered by immediate determinants like inadequacies in food, health, and care for infants and young children, especially in the initial two years of life. Underlying and basic determinants like socio-economic conditions, food security, hygiene, intervention like sanitation programs, and women’s empowerment can potentially improve nutrition [17]. Improving nutritional indicators requires investments in changing the determinants of poor nutrition, using a variety of efforts and policy instruments. In this study, we examine changes in the immediate determinants and nutrition-specific interventions to address those determinants and changes in the underlying determinants of nutrition.

**Figure 10:**
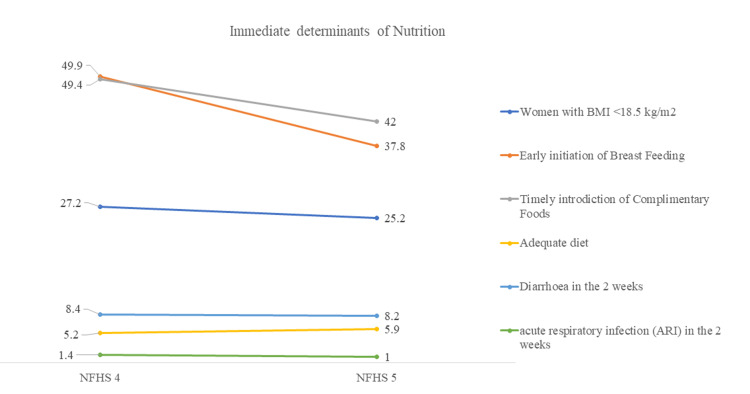
Changes in immediate determinants of nutrition in Gujarat, NFHS-4 and NFHS-5 The author has adopted the figure from NFHS-4 and NFHS-5 open-source data [11-15]. NFHS, National Family Health Survey

Changes in the immediate determinants of nutrition in Gujarat are mentioned in Figure 10. The prevalence of women with BMI (<18.5 kg/m^2^) declined from 27.2% in NFHS-4 to 25.2% in NFHS-5. Early breastfeeding initiation has reduced from 49.9% to 37.8%, but despite high rates of institutional deliveries, there is a scope for improvement. Timely introduction of complementary feeding for infants six months and older is of great concern, which declined from 49.4% to 42%. Only 5.9% of children between six and 23 months of age received an adequate diet. The proportion of children with diarrhea has slightly declined from 8.4% to 8.2%, and the proportion of acute respiratory infections (ARIs) declined from 1.4% to 1%.

**Figure 11:**
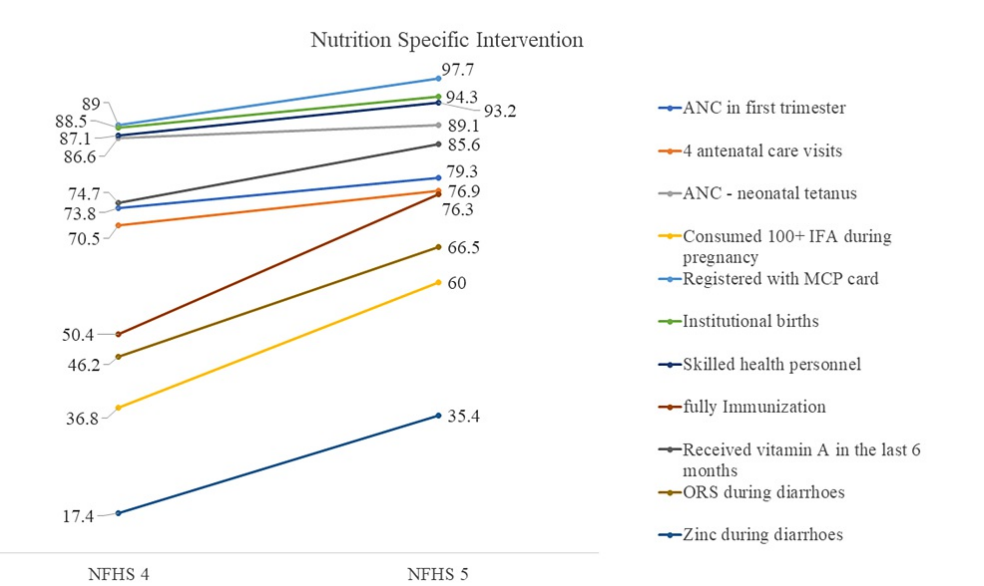
Changes in nutrition-specific intervention in Gujarat, NFHS-4 and NFHS-5 The author has adopted the figure from NFHS-4 and NFHS-5 open-source data [11-15]. NFHS, National Family Health Survey; ANC, antenatal care; IFA, iron and folic acid; MCP, mother and child protection; ORS, oral rehydration solutions

Changes in the coverage of nutrition-specific interventions in Gujarat are presented in Figure 11, which shows improvement in the coverage of all interventions since the last NFHS. During pregnancy, the proportion of women who received antenatal care (ANC) in the first trimester and at least four ANC visits improved substantially. The proportion of women who delivered in health facilities and whose delivery was assisted by skilled birth personnel also improved. IFA tablet consumption during pregnancy for 100 days or more increased from 36.8% to 60%. The proportion of fully immunized children increased from 50.4% to 76.3%, whereas children receiving vitamin A supplementation also increased. The children with diarrhea received oral rehydration salts, and zinc also increased from NFHS-4 to NFHS-5.

**Figure 12:**
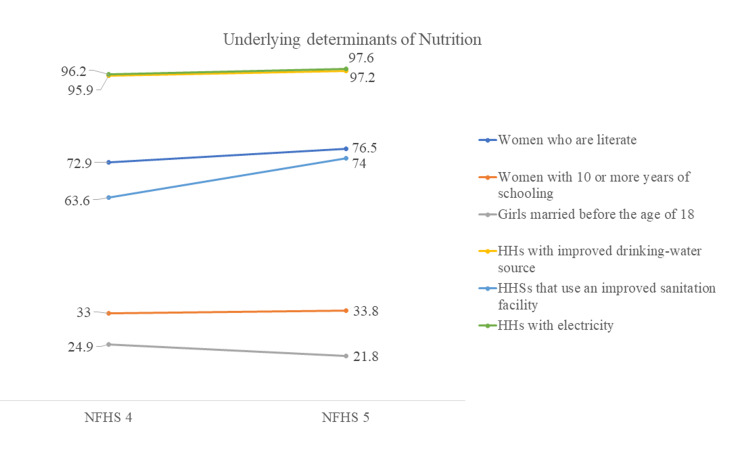
Changes in underlying determinants of nutrition in Gujarat, NFHS-4 and NFHS-5 The author has adopted the figure from NFHS-4 and NFHS-5 open-source data [11-15]. NFHS, National Family Health Survey; HHs, households

Changes in the underlying determinants of nutrition are presented in Figure 12. There has been a minimal increase in the proportion of literate women, but there remains much scope for improvement for women with 10 or more years of education. Early marriage in girls has dropped from 24.9% to 21.8%. Infrastructure has improved tremendously in Gujarat, as more than 95% of households have access to electricity and improved drinking water sources. The proportion of households using improved sanitation facilities has increased from 63.6% to 74%.

Inter-district variability in coverage of districts of Gujarat (determinants)

**Figure 13:**
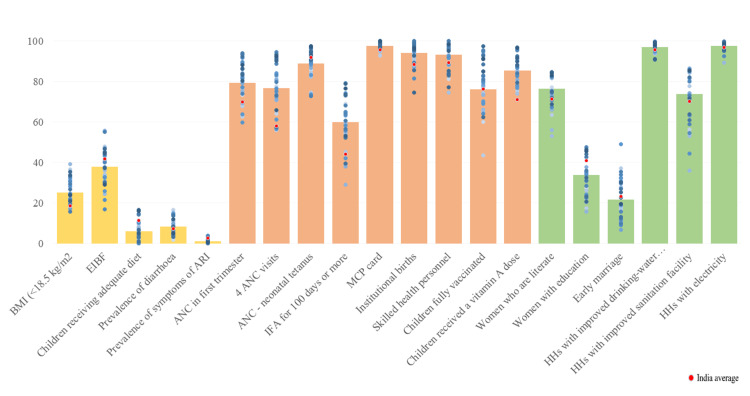
Inter-district variability of nutrition determinants of Gujarat, NFHS-5 The author has adopted the figure from NFHS-4 and NFHS-5 open-source data [11-15]. NFHS, National Family Health Survey; ARI, acute respiratory infection; ANC, antenatal care; IFA, iron and folic acid; MCP, mother and child protection; HHs, households

Overall, there have been improvements, and gaps among various determinants have been shortened to achieve the national mean. Determinants showing the lowest disparities are the prevalence of ARIs, households with electricity and improved drinking water source, and mothers receiving mother and child protection cards. Gujarat has performed well in determinants like BMI <18.5 kg/m^2^, four ANC visits, IFA for 100 days, institutional births, and birth assisted by skilled health professionals in surpassing the national mean. However, determinants like early initiation of breastfeeding, antenatal check-ups in the first trimester, IFA for 100 days, fully vaccinated children, literate women, educated women, early marriage, and households with improved sanitation show a high degree of inter-district variability (Figure 13).

Discussion

**Figure 14:**
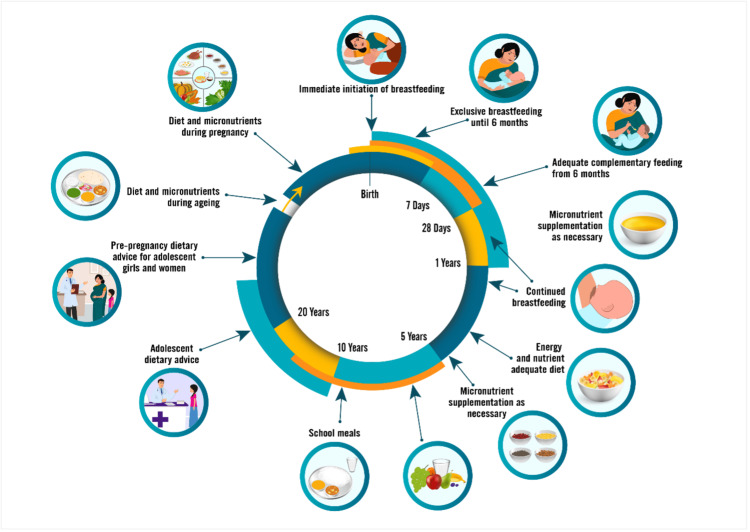
Improving nutrition around life-course Figure sourced from [22].

The child nutrition indicators have shown mixed patterns across states. Though the situation improved in many states/UTs, others have marginally deteriorated. The unlikely drastic changes have been observed in stunting and wasting quickly. Inadequate and improper nutrition in women is one of the most important determinants of stunting in their children. Between the NFHS-4 and NFHS-5, there has been a substantial improvement in the percentage coverage of nutrition-sensitive interventions, including women empowerment and maternal health services. Even with the implementation of these interventions, Gujarat has seen a rise in stunting and a marginal improvement in women's BMI. Undernourished children in Gujarat were disproportionately confined to low socio-economic sections in rural regions and some districts or geographical locations [11]. There was a glaring inter-district disparity in the rate of child stunting. As many as 24 districts had higher stunting rates than the national average (35.5%). This can be majorly attributed to primarily parental education and sanitation (Figure 13); such disparities observed could be the consequences of the partial reach of health policies. Household wealth, place of residence, and reproductive health care (ANC care, institutional delivery) may have attributed to stunting [11]. Many tribal-dominated districts like The Dangs, Narmada, Valsad, and Dahod were above the national average for child stunting. Surprisingly, Gujarat has been grappling with an increase in the prevalence of 10.6% (+1.1% from the last NFHS) for severely wasted children. Different observations are seen about the prevalence of stunting, wasting, and being overweight. The increasing prevalence of overweight in children (+2%) is of concern, as it adversely affects children's health and develops long-term chronic effects into adulthood [12]. Among all nutritional indicators, the prevalence of anemia is of much concern for Gujarat and poses a public health challenge. Surpassing the national average, Gujarat is corralling regarding anemia among children aged six to 59 months compared to 62.6% in NHFS-4, with an astounding increment to 79.7% in NFHS-5 (+17.1%). Whereas anemia in non-pregnant women increased to 65.1% from 55.1% (+10%), and in pregnant women, it increased to 62.6% from 51.3% (+11.3%). The prevalence of anemia is more than 60% across more than two-thirds of districts of Gujarat, among all specified age groups (children six to 59 months, women 15-19 years, reproductive age women 15-49 years, among non-pregnant women and pregnant women), which indicates a trend of an anemic individual going through the life cycle being anemic. Similarly, pregnant women consuming IFA tablets show more than 50% prevalence of anemia in two-thirds of the districts of Gujarat, suggesting that there can be a possibility of a type of anemia other than iron deficiency plaguing the women populace of Gujarat. The study has identified and categorized the districts of Gujarat state based on nutritional indicators (Figure 14) into top-priority, priority, average, and front-runner districts of Gujarat state. The top-priority districts require immediate actions to improve its indicator [18-21].

Gujarat compared to better-performing states

According to NFHS-5, 23 determinants directly or indirectly impact nutritional indicators. Some states like Kerala, Manipur, Mizoram, Punjab, and Arunachal Pradesh have most of their determinants performing better than the national average. For the state of Gujarat, special focus needs to be given to immediate determinants like early initiation of breastfeeding (37.8% for Gujarat compared to the national average of 41.8%, 66.7% for Kerala, 53.7% for Manipur, 60.1% for Mizoram, 53.1% for Punjab, and 52% for Arunachal Pradesh), children receiving solid or semi-solid food and breast milk, that is, complementary feeding (42% for Gujarat compared to the national average of 45.9%, 71.3% for Kerala, 78.9% for Manipur, 56.9% for Mizoram, 46.2% for Punjab, 48.4% for Arunachal Pradesh, and 57.4% for Sikkim) and children of six to 23 months receiving adequate diet (5.9% for Gujarat compared to the national average of 11.3%, 23.5% for Kerala, 19.6% for Manipur, 13.4% for Mizoram, 11.9% for Punjab, 22% for Arunachal Pradesh, and 22.3% for Sikkim). For nutrition-specific determinants, Gujarat has been making good strides alongside other states, only Kerala being the best-performing state in India. Gujarat performs better than the national average in nine out of 11 nutrition-specific determinants. Regarding underlying determinants of nutrition, 98.3% of women's literacy is seen in the state of Kerala, whereas Gujarat stands at 76.5%, marginally better than the national average. Among underlying determinants of nutrition, women with 10 or more years of schooling (33.8% for Gujarat compared to 41% national average) and women of age 20-24 years married before the age of 18 years (21.8% for Gujarat compared to 23.3% national average) have a substantial impact on nutritional indicators. Gujarat has scope for much improvement, focusing primarily on improving the nutrition-specific intervention and underlying and immediate determinants for better outcomes (Table 1).

**Table 1:** Categorization of district based on nutritional indicators The author has adopted the table from NFHS-4 and NFHS-5 open-source data [11-15]. NFHS, National Family Health Survey; SAM, severe acute malnutrition

	Children under five who are stunted (height for age %)	Children under five who are wasted (weight for height %)	Children under five who are severely wasted SAM (weight for height %)	Children under five who are underweight (weight for age %)	Children under five who are overweight (weight for height %)	Women whose BMI is below normal (BMI <18.5 kg/m^2^)	Women who are overweight or obese (BMI ≥25 kg/m^2^)	Children age six to 59 months (<11 gm/dL) (%)	Non-pregnant women (15-49 yrs) anemic (<12 gm/dL)	Pregnant women (15-49 yrs) (<11 gm/dL)	All women age 15-49 years who are anemic	All women age 15-19 years who are anemic
High-priority districts	Dahod, Patan, Chota Udepur	The Dangs, Tapi, Panchmahal	The Dangs, Panchmahal, Devbhoomi, Dwarka	The Dangs, Dahod, Narmada	Rajkot, Amreli, Vadodara	Dahod, Banaskantha, Tapi	Junagadh, Ahmedabad, Gandhinagar	Narmada, Panchmahal, Aravalli	Chota Udepur, Tapi, The Dangs	Aravalli, Bharuch, Narmada,	Chota Udepur, Tapi, Aravalli	Aravalli, Valsad, Narmada
Priority districts	Narmada, Aravalli, Panchmahal, Gir Somnath, Mahisagar, Vadodara, Tapi, Bharuch	Sabarkantha, Kheda, Gandhinagar, Bhavnagar, Aravalli, Navsari, Anand, Chota Udepur	Tapi, Sabarkantha, Surat, Porbandar, Chota Udepur, Dahod, Jamnagar, Morbi	Panchmahal, Tapi, Mahisagar, Chota Udepur, Aravalli, Anand Bharuch, Gandhinagar	Botad, Dahod, Surat, Surendranagar, Gir, Somnath, Devbhoomi, Dwarka, Sabarkantha, Chota Udepur	Aravalli, The Dangs, Panchmahal, Kheda, Anand, Chota Udepur, Mahisagar, Narmada	Jamnagar, Bhavnagar, Amreli, Rajkot, Porbandar, Vadodara, Botad, Kachch	Chota Udepur, Valsad, Dahod, Vadodara, Mehsana, Mahisagar, Kheda, Surat	Aravalli, Kheda, Narmada, Valsad, Dahod, Mahisagar, Vadodara, Bharuch	Panchmahal, Kheda, Chota Udepur, Tapi, Valsad, Ahmedabad	The Dangs, Kheda, Narmada, Valsad, Dahod, Mahisagar, Vadodara, Bharuch	Chota Udepur, Dahod, Mahisagar, Mehsana, Kheda, The Dangs, Surat, Vadodara
Average districts	Surendranagar, Banaskantha, Rajkot, Anand Gandhinagar, Valsad, The Dangs, Kachch, Junagadh, Kheda, Sabarkantha	Mehsana, Dahod, Surendranagar, Mahisagar, Devbhoomi, Dwarka, Botad, Surat, Banaskantha, Morbi, Bharuch, Jamnagar	Kheda, Mehsana, Mahisagar, Anand, Kachch, Gandhinagar, Narmada, Botad, Aravalli, Navsari, Valsad	Banaskantha, Navsari, Patan, Surendranagar, Mehsana, Sabarkantha, Vadodara, Bhavnagar, Kheda, Rajkot, Devbhoomi, Dwarka	Bharuch, Ahmedabad, Junagadh, Morbi, Porbandar, Anand, Jamnagar, Navsari, Panchmahal, Aravalli, Narmada, Patan	Bharuch, Patan, Kachch, Sabarkantha, Gir, Somnath, Mehsana, Surendranagar, Valsad, Devbhoomi, Dwarka, Navsari	Bharuch, Surat, Morbi, Mehsana, Devbhoomi, Dwarka, Valsad, Anand, Surendranagar, Navsari, Patan, Aravalli	The Dangs, Gandhinagar, Sabarkantha, Bharuch, Surendranagar, Tapi, Banaskantha, Anand, Porbandar, Rajkot, Patan	Mehsana, Panchmahal, Surat, Gandhinagar, Navsari, Sabarkantha, Anand, Rajkot, Patan, Banaskantha, Botad	Dahod, Banaskantha, The Dangs, Vadodara, Mehsana, Surendranagar, Patan, Surat	Panchmahal, Mehsana, Surat, Gandhinagar, Navsari, Sabarkantha, Anand, Ahmedabad, Rajkot, Banaskantha, Patan	Sabarkantha, Gandhinagar, Tapi, Rajkot, Navsari, Panchmahal, Bharuch, Ahmedabad, Banaskantha, Anand, Kachch
Front-runners districts	Navsari, Surat Ahmedabad, Amreli, Morbi Bhavnagar, Botad, Mehsana, Devbhoomi, Dwarka, Jamnagar, Porbandar	Amreli, Valsad, Narmada, Porbandar, Patan, Kachch, Vadodara, Gir, Somnath, Rajkot, Ahmedabad, Junagadh	Surendranagar, Banaskantha, Bharuch, Ahmedabad, Rajkot, Amreli, Junagadh, Patan, Bhavnagar, Vadodara, Gir Somnath,	Valsad, Ahmedabad, Kachch, Surat Botad, Morbi, Amreli, Gir, Somnath, Jamnagar, Junagadh, Porbandar	Kachch, Mahisagar, Kheda, Tapi, Bhavnagar, Mehsana, Banaskantha, The Dangs, Gandhinagar, Valsad	Gandhinagar, Morbi, Surat, Vadodara, Bhavnagar, Botad, Rajkot, Ahmedabad, Junagadh, Amreli, Jamnagar, Porbandar	Kheda, Gir, Somnath, Panchmahal, Sabarkantha, Tapi, Banaskantha, Narmada, Mahisagar, The Dangs, Chota Udepur, Dahod	Botad, Morbi, Navsari, Jamnagar, Junagadh, Ahmedabad, Amreli, Bhavnagar, Gir, Somnath, Kachch, Devbhoomi, Dwarka	Junagadh, Kachch, Surendranagar, Morbi, Jamnagar, Amreli, Gir, Somnath, Bhavnagar, Devbhoomi, Dwarka, Porbandar, Ahmedabad	Gir, Somnath, Gandhinagar, Junagadh, Mahisagar, Bhavnagar, Porbandar, Morbi, Sabarkantha, Devbhoomi, Dwarka, Botad, Kachch	Botad, Junagadh, Kachch, Surendranagar, Morbi, Jamnagar, Amreli, Gir, Somnath, Bhavnagar, Devbhoomi, Dwarka, Porbandar	Morbi, Botad, Patan, Jamnagar, Surendranagar, Junagadh, Porbandar, Bhavnagar, Devbhoomi, Dwarka, Gir, Somnath, Amreli

Implications and recommendations

Gujarat contributes tremendously to India’s overall burden of malnutrition. In the time of India’s commitment to global nutrition targets, it is urgent that Gujarat sets its nutrition target and accelerates the actions necessary to improve all forms of malnutrition. There are major challenges in several nutritional outcomes, particularly anemia. Anemia among children, women, and men has drastically increased in NFHS-5, which stands above the national average, and it is of great concern. The most worrisome indicator is the prevalence of anemia across the state of Gujarat. Not one but all indicators about the prevalence of anemia needs to be addressed promptly and effectively across the state. Since the last NFHS, Gujarat has seen improvements in most coverage of nutrition-specific interventions and underlying determinants. Although Gujarat has not created ripples in most of the nutrition indicators, there is a need for special attention to address the determinants contributing to anemia. A solution to tackle malnutrition in Gujarat needs to be initiated immediately.

The state shows the reverse trends in stunting, severe wasting, underweight, and overweight among under-five children. It indicates for strong multipronged strategy in Gujarat that considers all forms of malnutrition. Gujarat should invest in sustaining adequate conveyance of intervention about the first thousand days of life where coverage is quite well, while focusing on improving the coverage of the intervention that lags. On immediate determinants of Gujarat, special emphasis must be given to early initiation of breastfeeding, complimentary feeding practices, and children receiving adequate diet. On nutrition-specific intervention, we should focus on enhancing maternal care during pregnancy, that is, antenatal check-ups in the first trimester, and the increased current level of IFA consumption among pregnant women with more emphasis on sustaining the achieved progress on institutional births as well as delivery accompanied by skilled personnel. On underlying determinants, women’s education, early marriage, and sanitation require urgent attention.

Interventions are required to improve nutrition among women, starting early, essential for better pregnancy-related and early child health outcomes. This may have beneficial long-term effects on young girls and soon-to-be mothers; until then, iron deficiency is prevalent in the population. Public-health strategies such as iron supplements are necessary but insufficient to reduce childhood anemia. Instead, combining iron supplementation and food-fortification programs with efforts to reduce maternal anemia, family poverty, and food insecurity may yield optimal improvement in children’s hemoglobin levels [19]. The state needs to enhance its program's effectiveness and reach. It calls for proactive measures and scaling up of innovations required to address malnutrition and anemia and strengthen actions on healthy diets; maternal, infant, and young child nutrition; management of wasting; micronutrient supplementation; school feeding and nutrition; and nutrition surveillance, especially at inter-district levels. Advocacy of multisectoral actions is needed across life-course across all levels for better nutrition outcomes in the near future (Figure 14). Proper planning and implementation of existing schemes and policies with timely monitoring, evaluation, and evaluation of adequate funding can increase the efficiency of the program.

## Conclusions

This comparative study concluded that investing in overall nutrition should be Gujarat's priority. Special attention needs to be given to young children to prevent them from slipping into undernutrition. Gujarat should primarily focus on districts like Narmada, The Dangs, Tapi, Dahod, Panchmahal, Chotta Udaipur, and Aravalli as they suffer from multiple burdens, and it should address issues across the entire spectrum of potential determinants in these districts. The double burden of undernutrition and anemia can have dire consequences for Gujarat’s progress. The state has not created waves in most of the nutrition indicators. Hence, a need for special attention to addressing the determinants contributing to anemia and putting in place a solution to tackle malnutrition in Gujarat needs to be initiated immediately. It is high time to make nutrition a top investment priority in Gujarat.

## Appendices

**Table 2:** Nutritional status of children under five years The author has adopted the table from NFHS-4 and NFHS-5 open-source data [11-15]. NFHS, National Family Health Survey; SAM, severe acute malnutrition

	Children under five who are stunted (height for age %)			Children under five who are wasted (weight for height %)			Children under five who are severely wasted SAM (weight for height %)			Children under five who are underweight (weight for age %)			Children under five who are overweight (weight for height %)		
	NFHS-4	NFHS-5	Mean difference	NFHS-4	NFHS-5	Mean difference	NFHS-4	NFHS-5	Mean difference	NFHS-4	NFHS-5	Mean difference	NFHS-4	NFHS-5	Mean difference
Gujarat	38.5	39	0.5	26.4	25.1	-1.3	9.5	10.6	1.1	39.3	39.7	0.4	1.9	3.9	2
Ahmedabad	29.4	35.5	6.1	27.1	17.5	-9.6	11.5	7.4	-4.1	31	34	3	na	4.6	-
Amreli	37.8	35.3	-2.5	24.6	23.7	-0.9	6.4	7.2	0.8	31.7	30.6	-1.1	0.5	6.8	6.3
Anand	48.2	38.4	-9.8	21.7	28.6	6.9	7.4	10.9	3.5	41.3	46.6	5.3	0.7	3.8	3.1
Aravalli	50.6	47.1	-3.5	23.5	29	5.5	7	9.3	2.3	45.5	47.2	1.7	na	3.3	-
Banaskantha	40.7	39	-1.7	21.6	25.5	3.9	8.5	8.5	0	43.1	44.1	1	0.7	1.2	0.5
Bharuch	41.5	40.9	-0.6	29.4	24.5	-4.9	7.6	8	0.4	44.2	45.5	1.3	0	4.7	4.7
Bhavnagar	48.4	32.6	-15.8	26	29.6	3.6	7.9	6.3	-1.6	44.4	39.5	-4.9	na	1.8	-
Botad	38.9	32.2	-6.7	26.6	26	-0.6	9.7	9.6	-0.1	37.7	32.3	-5.4	na	5.8	-
Chota Udepur	43.8	48.6	4.8	16.3	28.4	12.1	5.8	13.8	8	39.1	48.1	9	na	4.8	-
Dahod	44.4	55.3	10.9	24.9	27.8	2.9	7.8	13.4	5.6	50.8	53	2.2	0.5	5.8	5.3
Devbhoomi Dwarka	27.9	30.2	2.3	31.3	26.1	-5.2	16.8	17.2	0.4	29.3	36.2	6.9	na	4.9	-
Gandhinagar	36.5	38.1	1.6	29	30.6	1.6	10.2	9.9	-0.3	42.7	44.6	1.9	0.3	0.5	0.2
Gir Somnath	27.9	44.4	16.5	30.4	18.5	-11.9	17.5	4.9	-12.6	27.2	30.3	3.1	na	5	-
Jamnagar	27.9	28.4	0.5	31.3	23.8	-7.5	16.8	12.5	-4.3	29.3	28.9	-0.4	na	3.6	-
Junagadh	27.9	37.3	9.4	30.4	17.3	-13.1	17.5	7.1	-10.4	27.2	26.4	-0.8	na	4.4	-
Kachch	40.8	37.5	-3.3	31.4	20.2	-11.2	15.5	10.6	-4.9	39	33.4	-5.6	1.6	2.6	1
Kheda	45.5	37.3	-8.2	27.2	30.9	3.7	7.1	12.1	5	48.1	39.5	-8.6	na	2.5	-
Mehsana	40.5	31	-9.5	25.3	28.2	2.9	11.6	12	0.4	41.9	41.4	-0.5	1.3	1.5	0.2
Mahisagar	43	43.4	0.4	31.8	26.2	-5.6	10.7	12	1.3	45.2	49	3.8	na	2.6	-
Morbi	38.2	32.9	-5.3	25.6	25.2	-0.4	6.6	12.5	5.9	38.7	32.3	-6.4	na	4	-
Narmada	47.4	47.2	-0.2	35.8	23	-12.8	12.7	9.9	-2.8	53.6	52.8	-0.8	1	3.1	2.1
Navsari	38.9	36.8	-2.1	26.8	29	2.2	5.7	9.3	3.6	37.4	43.6	6.2	0	3.4	3.4
Panchmahal	40.4	47.1	6.7	36.3	35.7	-0.6	14.2	19.7	5.5	42.3	51.9	9.6	na	3.4	-
Patan	37.6	50.5	12.9	24.5	20.9	-3.6	9.2	6.5	-2.7	38.4	42.3	3.9	1.2	3.1	1.9
Porbandar	22.6	18.2	-4.4	25.4	21.8	-3.6	10.6	14.2	3.6	27.7	25.5	-2.2	2.3	3.9	1.6
Rajkot	30.9	38.9	8	23.4	17.6	-5.8	3.7	7.3	3.6	31.4	37	5.6	na	8.1	-
Sabarkantha	50.6	37	-13.6	23.5	33.1	9.6	7	16.6	9.6	45.5	41	-4.5	na	4.9	-
Surat	30	36.1	6.1	26.2	26	-0.2	8.1	14.7	6.6	36.1	32.5	-3.6	2.9	5.3	2.4
Surendranagar	45.5	39.2	-6.3	27.7	27.1	-0.6	9.5	8.8	-0.7	45.9	41.8	-4.1	na	5.2	-
Tapi	35.9	41.7	5.8	35.8	36.6	0.8	9.6	17.1	7.5	42.2	51.8	9.6	1.2	1.9	0.7
The Dangs	48.1	37.6	-10.5	43	40.9	-2.1	18.9	22.2	3.3	60	53.1	-6.9	2.3	1.1	-1.2
Vadodara	43.8	42.3	-1.5	16.3	20.1	3.8	5.8	5.2	-0.6	39.1	39.9	0.8	na	6.4	-
Valsad	43.3	37.8	-5.5	30.3	23.2	-7.1	11.9	9.1	-2.8	41.9	34.8	-7.1	3.9	0.3	-3.6

**Table 3:** Nutritional status of adults (age 15-49 years) The author has adopted the table from NFHS-4 and NFHS-5 open-source data [11-15]. NFHS, National Family Health Survey

	Women whose BMI is below normal (BMI <18.5 kg/m^2^ )			Women who are overweight or obese (BMI ≥25 kg/m^2^ )		
	NFHS-4	NFHS-5	Mean difference	NFHS-4	NFHS-5	Mean difference
Gujarat	27.2	25.2	-2	23.7	22.6	-1.1
Ahmedabad	21.5	19.4	-2.1	30.7	30.1	-0.6
Amreli	18.3	18.3	0	28.2	29.1	0.9
Anand	36	32	-4	19.4	20.9	1.5
Aravalli	37.1	34.4	-2.7	12.9	18.1	5.2
Banaskantha	38.4	36.7	-1.7	10.6	11.7	1.1
Bharuch	31.3	30.3	-1	23.7	25.5	1.8
Bhavnagar	21.5	20.8	-0.7	28	29.4	1.4
Botad	21.5	19.6	-1.9	29.4	27.3	-2.1
Chota Udepur	29.1	30.9	1.8	22	7.6	-14.4
Dahod	44.1	39.1	-5	9.8	6.5	-3.3
Devbhoomi Dwarka	19.5	22.4	2.9	29.4	24.1	-5.3
Gandhinagar	30.5	21.2	-9.3	22.1	29.5	7.4
Gir Somnath	16.9	24.8	7.9	25.5	17.3	-8.2
Jamnagar	19.5	17.3	-2.2	29.4	29.5	0.1
Junagadh	16.9	18.7	1.8	25.5	33.4	7.9
Kachch	23.7	28.1	4.4	19.9	25.7	5.8
Kheda	38.5	32.2	-6.3	18	17.8	-0.2
Mehsana	26.8	24.4	-2.4	22.7	24.3	1.6
Mahisagar	42.6	30.7	-11.9	14.1	9.4	-4.7
Morbi	21.6	21.2	-0.4	28.4	24.6	-3.8
Narmada	44.1	30.7	-13.4	11.4	11.1	-0.3
Navsari	29.5	21.8	-7.7	23.1	18.7	-4.4
Panchmahal	46.8	33.1	-13.7	10.1	16.1	6
Patan	30.2	29.3	-0.9	17	18.3	1.3
Porbandar	14	15.8	1.8	24.8	27.8	3
Rajkot	17.4	19.5	2.1	36.1	28	-8.1
Sabarkantha	37.1	26.6	-10.5	12.9	15.5	2.6
Surat	18.4	21	2.6	34.5	25.1	-9.4
Surendranagar	25.7	24.1	-1.6	20.6	20.7	0.1
Tapi	43.4	35.4	-8	8.8	12.6	3.8
The Dangs	44	33.7	-10.3	4	8.1	4.1
Vadodara	29.1	20.9	-8.2	22	27.6	5.6
Valsad	22.9	23.8	0.9	24.8	21.3	-3.5

**Table 4:** Prevalence of anemia The author has adopted the table from NFHS-4 and NFHS-5 open-source data [11-15]. NFHS, National Family Health Survey

	Children aged six to 59 months (<11 gm/dL %)			Non-pregnant women (15-49 years) anemic (<12 gm/dL)			Pregnant women (15-49 years, <11 gm/dL)			All women aged 15-49 years who are anemic			All women aged 15-19 years who are anemic		
	NFHS-4	NFHS-5	Mean difference	NFHS-4	NFHS-5	Mean difference	NFHS-4	NFHS-5	Mean difference	NFHS-4	NFHS-5	Mean difference	NFHS-4	NFHS-5	Mean difference
Gujarat	62.6	79.7	17.1	55.1	65.1	10	51.3	62.6	11.3	54.9	65	10.1	56.9	69	12.1
Ahmedabad	76	72	-4	62.9	17.5	-45.4	62.7	70.1	7.4	62.9	63.7	0.8	na	67.3	-
Amreli	74.5	72	-2.5	55.8	50.3	-5.5	64.1	-	-	56	50	-6	58.3	47	-11.3
Anand	59.7	78.4	18.7	50.7	66.6	15.9	42.6	-	-	50.4	66.4	16	46.8	64.2	17.4
Aravalli	72.5	89.5	17	67.7	77.1	9.4	52.3	82.6	30.3	67.2	77.3	10.1	na	87.7	-
Banaskantha	57.2	79	21.8	50.1	59.6	9.5	49.1	68.6	19.5	50	60	10	45.3	65.8	20.5
Bharuch	56.8	81	24.2	51.8	71.6	19.8	55.6	77.2	21.6	52	71.8	19.8	59.9	69.8	9.9
Bhavnagar	69.2	71.5	2.3	54.1	49.1	-5	53.6	56.2	2.6	54	49.4	-4.6	na	52	-
Botad	72.6	75.5	2.9	58.5	59.1	0.6	58.2	48.5	-9.7	58.5	58.8	0.3	na	60.3	-
Chota Udepur	54.3	87.7	33.4	49.3	79.1	29.8	48.2	72.1	23.9	49.2	78.9	29.7	na	80.3	-
Dahod	58.9	87.2	28.3	55.9	75.4	19.5	64.6	69.4	4.8	56.6	75.1	18.5	56.9	79.7	22.8
Devbhoomi Dwarka	75.7	66.7	-9	64.2	48.8	-15.4	56.7	49.1	-7.6	63.8	48.8	-15	na	50.9	-
Gandhinagar	73.7	81.2	7.5	66.3	69.2	2.9	na	57.4	-	65.8	68.8	3	64.4	72.8	8.4
Gir Somnath	77.1	68.9	-8.2	60.5	49.6	-10.9	39.4	57.7	18.3	59.7	49.9	-9.8	na	50.9	-
Jamnagar	75.7	75	-0.7	64.2	50.6	-13.6	56.7	na	-	63.8	50.2	-13.6	na	60.1	-
Junagadh	77.1	74.7	-2.4	60.5	58.8	-1.7	39.4	57.3	17.9	59.7	58.8	-0.9	na	55.5	-
Kachch	81.4	68.6	-12.8	63	58	-5	49.8	43.1	-6.7	62.5	57.5	-5	69.3	62.6	-6.7
Kheda	53.7	85.1	31.4	54.8	76.2	21.4	46.1	72.9	26.8	54.5	76.1	21.6	na	77.6	-
Mehsana	77.8	86	8.2	62.2	69.5	7.3	56.9	62.9	6	61.9	69.3	7.4	63.1	77.7	14.6
Mahisagar	52	85.9	33.9	52.5	73.2	20.7	51.6	56.5	4.9	52.5	72.5	20	na	79.4	-
Morbi	67.2	75.3	8.1	57.1	50.7	-6.4	55.6	50.1	-5.5	57.1	50.7	-6.4	na	62	-
Narmada	53.6	93.2	39.6	55.5	75.9	20.4	58.2	75.6	17.4	55.6	75.9	20.3	52.4	83	30.6
Navsari	51.9	75.3	23.4	51.9	68.9	17	na	na	-	52.1	68.7	16.6	48.4	72.6	24.2
Panchmahal	50.2	91	40.8	50.2	69.5	19.3	57	74	17	50.4	69.8	19.4	na	71.7	
Patan	67.2	76.2	9	59	59.8	0.8	69.4	60.1	-9.3	59.6	59.8	0.2	60	60.2	0.2
Porbandar	70.8	77.9	7.1	59.4	47.4	-12	53.7	54.9	1.2	59.2	47.6	-11.6	63.8	52.4	-11.4
Rajkot	57.6	77	19.4	52.8	60.6	7.8	46.3	na		52.6	60.5	7.9	na	72.7	
Sabarkantha	72.5	81.1	8.6	67.7	67.9	0.2	52.3	49.4	-2.9	67.2	67.3	0.1	na	73.8	
Surat	42.3	83.6	41.3	39.4	69.3	29.9	29	58.7	29.7	39	69	30	39.9	76.2	36.3
Surendranagar	76.8	81	4.2	61.3	55.4	-5.9	64.9	60.8	-4.1	61.5	55.6	-5.9	na	56.9	
Tapi	49.5	80.7	31.2	54.3	77.6	23.3	na	72		54.4	77.4	23	57.8	72.8	15
The Dangs	74.1	82.4	8.3	72.6	77.6	5	66.5	66.8	0.3	72.2	77.2	5	77.8	77.1	-0.7
Vadodara	54.3	86.4	32.1	49.3	72.6	23.3	48.2	65.9	17.7	49.2	72.3	23.1	na	74.3	
Valsad	50.4	87.6	37.2	50.8	75.8	25	53.6	71	17.4	50.9	75.7	24.8	57.7	85.8	28.1
